# Quality of health news disseminated in the print media in developing countries: a case study in Iran

**DOI:** 10.1186/1471-2458-12-627

**Published:** 2012-08-09

**Authors:** Mahnaz Ashoorkhani, Jaleh Gholami, Katayoun Maleki, Sima Nedjat, Jalaledin Mortazavi, Reza Majdzadeh

**Affiliations:** 1 Knowledge Utilization Research Centre (KURC), Tehran University of Medical Sciences, Tehran, Iran; 2 Knowledge Utilization Research Centre (KURC), School of Public Health, Tehran University of Medical Sciences, Tehran, Iran

## Abstract

**Background:**

Mass media play an important role in keeping people up-to-date with the latest health news. This study aims at investigating the quality of health news disseminated in the print media, its course of production and factors affecting its quality.

**Methods:**

In the quantitative section of the study, 410 health-related news items, published during a six-month span in the Iranian public press, underwent content analysis. In the qualitative section, focus group discussions were held with journalists, editors-in-chief and news gatekeepers.

**Results:**

The quantitative phase showed that 18% of the news articles were not fit for dissemination in public. The qualitative phase illustrated that multiple factors at various levels affect the quality of news, namely poor knowledge, inadequate motivations and context-related barriers.

**Conclusions:**

The quality of health news reporting is not desirable. Educational interventions need to be carried out to raise awareness among researchers and journalists. Also, certain steps should be taken to increase motivations and strengthen infrastructures, including designing guidelines and monitoring news.

## Introduction

Mass media is an important and influential element in the knowledge transfer and dissemination process [1], playing an important role in conveying scientific information to people and policy makers [2]. Health research findings are always highlighted by the media and rarely a day passes by where there is no medical and pharmaceutical news coverage [3].The public are very interested in health related news and up-to-date knowledge, seeking information on diseases, their prevention, diagnosis and treatment, nutrition, medications and other factors related to their health [4]. Research in various countries, shows health news is very popular and that it is the third most covered topic in the evening news. The role of the media in influencing public opinion and changing behavior is undeniable [1,5]. Health news changes people’s perspective and behavior toward health, and in some sparks curiosity in the topic discussed [6]. The influence of the media goes as far as some patients changing their mode of treatment [7]. In a related study, conducted by the American ‘National Health Council’ in 1998, it was observed that 75% of the people receive health news via the media (40% from the TV, 35% - by magazines or journals, 16% from newspapers, and 2% through the internet) [6]. No doubt greater access to the internet in recent years has changed the aforementioned percentages, but it is worth mentioning that Bartlett also concluded that newspapers are an important source of information on medical research findings [8]. Print media such as newspapers are among the primary sources of health news for both clinical specialists and the public [9-11].

Observations show that the media usually exaggerate the benefits of medications but under-report their side-effects and costs [12]. Some reports even contain wrong and potentially dangerous information which can harm those who trust them [13]. Publishing news on specific health interventions can be as harmful as it is beneficial, which should really come as no surprise, inasmuch as they give false hope to target audiences by sensationalizing and spotlighting the matter for latent professional and competitive reasons [3].

To the best of our knowledge, only so many studies in English have been published in peer reviewed journals on the quality of health news published in Iran or Middle Eastern countries, so we took it upon ourselves to conduct this study. First, we evaluated the quality of news published in the press and then identified the factors affecting such quality.

## Methods

The study consisted of two sections: quantitative and qualitative. The quantitative section was conducted in a 6 month period in which health related news published in the public press underwent content analysis for assessing the scientific quality of their sources. Among the Persian newspapers published in the country, 68 newspapers that had internet websites and had archived their daily news were selected. Newspapers lacking a health column were excluded from the study and eventually 21 were included.

Four hundred and ten news items were extracted and reviewed against a checklist. The checklist included the news topic, the name of the news agency, person or organization giving the news, and any reference to the research article published in peer reviewed journals.

Among these, 58 news items had referred to the research articles as their source. Two persons independently searched for the sources in Google Scholar and PubMed. Twenty nine articles were accessible. The 29 extracted research articles were then assessed for their quality, independently by two epidemiologists, using the 'Critical Skills Appraisal Programme' tool [14]. This tool was designed for assessing the internal and external validity of research reports and is widely used in quality assessments.

Since we needed the source of the news content and it was not mentioned in 352 of them, we turned to relying on experts' opinions for judgment on the quality of the remaining news items. Here, we thought we should either ask a variety of experts, and/or, select the topic which was most frequent among the news items extracted.

Hence, to examine the quality of those news items that had ‘not’ referred to a relevant article, nutrition was chosen as the most frequent news topic (77 out of 410). At this stage the news items were assessed for their quality and appropriateness for publication in mass media. For this, two nutrition experts independently went through the news items and examined their quality. Cases of disagreement were settled upon a third party’s opinion-a senior nutrition professor. The two questions used to examine nutrition news covered the following topics:

i. Presence of supporting evidence: reviewers were asked to specify which of the following options held true for the news: 1) this topic has been mentioned in reference textbooks, 2) the content of this news has been published in the form of a research article, 3) there are certain research articles in this field, but they have not been approved yet, 4) there are contradictory results in this field, and 5) there is no evidence on this matter.

ii. Scientific accuracy: these options were used to question the scientific accuracy of the news items: 1) It has been proven scientifically, 2) It may be correct, but has not been scientifically proven yet, 3) This is a matter of debate, and 4) It is scientifically incorrect.

The qualitative section of the study explored the process of production, selection and dissemination of health news and the factors affecting its scientific quality.

In this phase two focus group discussions and two in-depth interviews were held with 14 media representatives including journalists, editors-in-chief and members of the ‘Health Media Policy-Making Council’. The interviewer was a PhD epidemiologist familiar with the topic at hand, and a note-taker was present too. Interviewees were selected through purposive sampling. Each interview lasted an hour long, and the Focus Group Discussions (FGDs) lasted 2 hours. They were audio-recorded upon consent and consequently transcribed verbatim. The sessions were held either at the interviewees’ workplace at their request, or, the research team’s center. No one refused to be interviewed. The interviews and discussions’ manuscripts were fed into the 'Open-Code' software and studied through thematic analysis. Coding was done by two independent individuals and thereon 150 lines from one interview and another 150 lines from one FGD were chosen to examine the reliability and inter-rater agreement. The codes and categories allocated to each topic were compared with each other. The inter-rater agreement was 89%. The final codes were chosen in a session held by the research team.

This study has the approval of Tehran University of Medical Sciences’ Ethical Committee which abides by the Helsinki Declaration.

## Results

### Quantitative section

In the quantitative section, as mentioned earlier, the news items were classified into two groups: the first group consisted of news items that had referred to a research article, and the second one included news items that had ‘not’ referred to a research article. In the second group the most frequent topic, i.e. nutrition news was chosen to be examined as the representative.

In the first group, the study design of each article was studied. Two of them were systematic reviews, 10 were interventional, 12 were observational and 5 were basic science studies. Eighteen (62.1%) were of appropriate scientific quality level and 11 (37.9%) were weak. The news was also checked for its consistency with the research articles results. Four (13.8%) were completely consistent with the research articles results, 11 (37.9%) were highly consistent, 6 (20.7%) were somewhat consistent, and 8 (27.6%) were not consistent with the research findings. In the second group, 71 nutrition news items were examined by experts in this field. News that qualified for options 4 or 5 (mentioned in the methodology section: “there are controversial results in this field” and “there is no evidence available for this topic”) for supporting evidence, and qualified for options 3 or 4 of scientific accuracy (“it is a matter of debate”, it “is scientifically incorrect”) were considered to be unfit for dissemination. These cases comprised 10 out of 71 cases of nutrition news.

A summary of the study’s quantitative findings is presented in Table 1. On the whole, if we consider the 29 news items of the first group as representative of this group, and the 71 nutrition news items as representative of the second group, we may conclude that 18% of the news was not fit for public dissemination.

**Table 1:** Examination of news for its quality to be publicly announced

	** *The status of research evidence in disseminated news* **	** *Number of news items* **	** *Number of documents reviewed* **	** *Unfit for publication* **
				**Number (Percent)**
**1**	Referral to the research article, including its particulars	58	29	8 (27.6)
**2**	Referral to the research article, excluding its particulars	183	31*	7(22.6)
**3**	No referral to the research study	169	40*	3(7.5)
**Total number**		410	100	18 (18.8)

*Only nutrition news was examined by nutritionists (explained in detail in the text).

### Qualitative section

The results of the qualitative phase revealed two primary categories: ‘description of the process of production, selection and dissemination of news’ and ‘factors affecting the quality of news’. Below each category is described, and verbatim quotes from FGDs are included in italics to exemplify participants' comments.

1) Description of the process of production, selection and dissemination of news

Journalists have recognized a specific set of criteria and values for the production and selection of news content. From their perspective, news values include: bizarreness or rarity of news, the novelty and appeal, significance, proximity, and universality of news, and reputation and standing of the person announcing the news. Apart from the above general principles, in some units certain guidelines are observed by journalists in the news production process. The news prepared by journalists are checked and selected by the editor-in-chief or gatekeeper in the editorial section. It may be merged with other news or some material may be added or removed which may compromise the accuracy or content of the news. In addition to news criteria and values exercised by journalists, there are other criteria affecting the selection of news by editors, editors-in-chief and gatekeepers in the media, such as national archives, health priorities, daily events, emergencies etc. However, according to some of the participant editors, in Iran there are no standard or specific guidelines for news selection by editors, explaining why news is sometimes selected on the basis of personal taste.

2) Factors affecting the scientific quality of news

"*“Having a guideline of whatsoever quality is better than not having one at all.” (Member of the ‘Health Media Policy-Making Council’- 1)*"

"*“If we have 5 news items of equal weight, we will give priority to the news that is more eye-catching because more target audiences are absorbed.”(Editor-2)*"

In effect all participants believed that not all health news produced and published are qualified and accurate enough for public announcement. Different factors at various levels affect the accuracy of news. These factors can be classified into inadequate knowledge, inadequate motivations and context-related barriers.

a) Inadequate Knowledge

Some journalists believed that journalists do not have enough information in the field of health knowledge, and the dearth of specialized health journalists hinders the production of quality health news. In the same context, lack of required knowledge, concentration and accuracy in translation skills often leads to certain changes in the news content.

"*“Being familiar with health knowledge seems a requisite for people in this field, but this mechanism hasn’t been designed in our country yet.” (Journalist-5)*"

"*“At times foreign news is translated badly because of the translator’s lack of knowledge in the health domain.”(Journalist-2)*"

Gatekeepers’ knowledge has been mentioned as another important factor in monitoring the quality of news. Some editors recognized having adequate knowledge on the general principles of research as helpful, while many editors do not have sufficient knowledge in this field.

"*“Perhaps our biggest problem arises when we don’t have sufficient knowledge [on research]. The news editor doesn’t have a good grip over the topic.”(Editor- 4)*"

"*“The number of individuals with a grip over the study components like design, methodology etc., and quality appraisal skills is small.”(Editor-2)*"

Researchers who pass on health research results to journalists and news agencies are also unaware of reporting criteria and techniques, which is considered another common problem. Hence, researchers’ styles of writing is inappropriate for dissemination in the media, and journalists create certain changes in the news content to make them more appealing, but this might not exactly be in line with the concept the researcher has in mind. At this stage the content of the news may lose its original intent and accuracy.

b) Inadequate motivation

Time, circulation and sales matter to newspapers. Producing appealing news in a limited time period is of paramount importance to newsmen. With little alteration journalists familiar with news principles and journalistic techniques can turn unimportant news topics into headlines. Such changes often result in changing the original content of the news that greatly affects its quality.

"*"Some people and organizations have special ties with certain journalists or news agencies. Because of journalistic favoritism, news related to a certain person or group is covered differently, or differs even in content."(Journalist- 1)*"

A similar phenomenon happens in the domain of researchers cooperating with the press. Journalists and editors believed researchers played an influential role as one of the sources of news. According to them, sometimes competition among researchers leads to the early release of news or even its unreal and incorrect dissemination.

"*“Studies that are being conducted on animals should not be announced to the public. However, because of competition among researchers or institutes….the early announcement of news leads to its renouncement afterward. This way people lose trust in the media.”(Journalist-2)*"

On the other hand, researchers do not have adequate motivations toward collaborating with the media.

"“*For certain reasons, such as lacking a media perspective and financial issues etc., academics are not interested in cooperating with the media and acting as consultants for checking the authenticity of the health news.”(Journalist- 6)*"

Further, economic issues were among media's significant challenges. Currently, many research centers and companies use media consultants. The latter is done for marketing. Financial ties between pharmaceutical companies and researchers pave the way for advertising pharmaceutical products. These ties, in turn, lead to the concealment of adverse effects of drugs and to an exaggeration of their benefits.

c) Context-related barriers

"*"At times pharmaceuticals arrange meetings with physicians and researchers beforehand and convince them to announce news that would approve and promote their products."(Journalist-5)*"

"*"Potentially, 12% of the world drug market is in the Middle East… and our country is a potential target."(Editor-3)*"

Mass media organizational policies influence the quality of news. These policies demand that the health news be delivered to the society. They also demand that the country's scientific research achievements be announced to the public:

"*"Big projects are a source of pride for the nation, and people expect to be given good coverage."(Editor-3)*"

However, Journalists and editors-in-chief believed this exploitation of health news could be harmful, and lead to omission of results or exaggeration of the news. At times they are compelled to publish these kinds of news stories, the quality of which they themselves doubt.

According to these participants, the reputation of the person reporting the news and his/her socio-political position also influences them to publish the news.

"*"At times the reputation of the person announcing the news is even bigger than that of the gatekeeper's. When the health minister announces certain news the gatekeeper cannot prevent its dissemination."(Member of the ‘Health Media Policy-Making Council’-2)*"

"*“Politicizing scientific news exaggerates some topics.” (Journalist-6)*"

"*“At times the limits prevent the editors and editors-in-chief from improving or criticizing the news.” (Editor-3)*"

## Discussion

Using both quantitative and qualitative approaches to data collection, this study aimed to examine the quality of health news published and the factors affecting their publication. The results of the quantitative section revealed that 18% of the news did not scientifically qualify for public dissemination. The qualitative data illustrated that multiple factors at different levels affect the quality of news. From the journalists and editors-in-chief's perspectives they were level of research, health awareness and also difficulties in translation of documents. The factors that influenced research quality from the researchers’ side were lack of familiarity with journalistic techniques, not having a clear perspective towards the media, competition among researchers and also ties between researchers and commercial or pharmaceutical companies. Context-related barriers e.g. organizational policies, affected both sides.

The generalizability of the results of examining 100 out of 410 news articles can be considered the limitation of this study. However, it is safe to say that selection bias does not appear to be a problem. We argue that because the 29 research articles accessed were from reputable journals, enlisted in Tehran University of Medical Sciences digital library, the full texts that were not accessible are not expected to be of higher quality than those found. Therefore, not only is 18% not an overestimate, but also the percentage may even be considered an underestimate, because, in this study we only examined the quality of news evidence. No doubt, if more than one criterion, i.e., examining the quality of evidence, such as those recommended by Media Doctor (was taken into consideration, assessment of advantages and disadvantages, and conflicts of interests) [15], then the percentage of news unfit for public dissemination would exceed 18%, but the purpose of this study was to study the quality of evidence alone.

According to Table 1, 27.6% of the news articles that had cited a peer-reviewed journal did not qualify for publication. This proportion was 7.5% in the news that had not referred to the research articles at all. It seems that journalists do not appropriately reflect evidence from research articles. They disseminate their own impressions of the subject, while they cite the research article.

The two factors identified at the level of researchers and journalists in the qualitative section of the study were inadequate knowledge and motivation. Studies conducted elsewhere also report that the selection and creation of news is inevitably influenced by journalists’ knowledge, ideology, interests, and factors such as driving the viewer or reader’s attention, practical limitations and political atmosphere [16-19]. Another aspect of health news is that even though journalists may be professional in the field of news, they have insufficient knowledge in health sciences [20,21]. Other identified factors are health news translators’ inadequate knowledge of health topics and mastery of the language, competition among journalists, and absence of specialized health journalists. Many of the news items published are based on translations of literature and articles, therefore it is better to have translators familiar with medical and health sciences, national health policies and priorities, to prevent unscientific translations and to promote appropriate news selection [21]. In the qualitative section of our study too, some of the journalists expressed concern over their ability to understand, interpret and translate English into their own language. Therefore focusing on this aspect will help improve the quality of health news. Journalists’ time constraints and limited knowledge have been stated as the most important barriers in promoting health journalism by Larsson. Competition, difficulties in understanding scientific terminologies, finding and using references and economic issues are other barriers she underscored [20]. Even though we also identified economic factors to be influential in news selection, which is a similar finding to that in developed countries, its nature and severity may be different. Hochman et al. were concerned with pharmaceutical company funding of medical research as an influential factor; in many instances the journalists are not aware of a pharmaceutical company sponsor [22]. Moynihan et al. believed that the benefits of medications are exaggerated or highlighted and their side effects are sidestepped and that such behaviors are the results of financial ties with drug manufacturers [12].

Inadequacy of editors and gatekeepers awareness regarding research methodology and critical appraisal is another reason identified in our study. These individuals are the ones who have ultimate authority over what gets published, so it is necessary for them to acquire such knowledge.

On the other hand most researchers do not possess journalistic skills, and do not pay attention to the appeal of the title. That is why journalists alter the content of the news and its accuracy to make the news appealing. Training researchers or directly linking them to journalists in the news production process can prove helpful.

The third factor identified was context-related barriers. Certain steps taken in other countries have strengthened their infrastructures. For example, in 1991 the ‘Press Complaints Commission’ in UK designed a framework and obligated it for the press. Subsequently, if incorrect material is published, immediate notification and fair reporting should take place [3]. Also, principles for producing and disseminating health news have been designed by ‘The Association of Health Care Journalists’ (AHCJ), an independent association compromising 750 members [23]. Such measures have not entered Iran’s health news arena and we have yet to take steps toward standardizing the procedure. Literature shows that pressurizing alone does not help in implementing these principles; educating journalists and editors in the field is a more effective approach [23]. Our results also show that educating journalists may prove beneficial. In Iran the Masters degree in Medical Journalism was initiated in the universities in 2009. But, only MD degree holders are admitted, and, by the time this paper was written (2011) the admittance capacity was three per year only [24]. Training researchers and health journalists and monitoring health news production have been recommended elsewhere too. Entwistle et al. have proposed solutions for improving the current situation through creation of health news knowledge networks under which health journalists, editors-in-chief, clinical service providers, researchers and consumer right activists can act unanimously. Among other solutions are: commitment to establishment of medical report review committees for quality monitoring, pointing out possible flaws and shortcomings of research, and educating news producers to critically appraise medical news [3,20,23]. Already, certain organizations comprising specialists have been created around the world to monitor the news disseminated. For example ‘Media Doctor’ in Australia and Canada and ‘Health News Review’ in USA review health news reports on the basis of certain criteria and display only those news items that are eligible in their sites [25]. Developing and strengthening such sites is one way of overcoming the current problems in the field.

To wrap it up, the results of our study imply that developing countries need to create infrastructures in news producing organizations; to strengthen the quality of health news, to design guidelines and to foresee necessary procedures for capacity building among the concerned manpower.

## Conclusions

The quality of the health news published in print media is not desirable. The following three contributing factors have been identified:

1- Inadequate knowledge

2- Inadequate motivation

3- Context-related barriers

Hence, education alone will not suffice without the appropriate infrastructures. Therefore creating a news production manual, culture-building at the level of researchers and media personnel (to increase motivations for producing quality news), and creating organizations specialized in monitoring the quality of news seem necessary.

## Competing interests

The authors declare that they have no competing interests.

## Authors’ contributions

RM elaborated the idea of the study. RM and JG designed the study. MA, SN, KM and JM participated in the data gathering. MA and JG performed the statistical analysis of the quantitative part. SN assisted in the qualitative analysis of the study. RM, MA and JM contributed in interpretation of the results. MA, RM and KM participated in the manuscript writing. All authors read and approved the final manuscript.

## Pre-publication history

The pre-publication history for this paper can be accessed here:

http://www.biomedcentral.com/1471-2458/12/627/prepub
